# Zn-Mg Biodegradable Composite: Novel Material with Tailored Mechanical and Corrosion Properties

**DOI:** 10.3390/ma12233930

**Published:** 2019-11-27

**Authors:** Jiří Kubásek, Drahomír Dvorský, Jaroslav Čapek, Jan Pinc, Dalibor Vojtěch

**Affiliations:** 1 Department of Metals and Corrosion Engineering, Faculty of Chemical Technology, University of Chemistry and Technology, Prague Technická 5, Dejvice, 166 28 Prague 6, Czech Republic; dvorskyd@vscht.cz (D.D.); Vojtechd@vscht.cz (D.V.); 2 Department of Functional Materials, Institute of Physics of the Czech Academy of Sciences, Na Slovance 1999/2, 182 21 Prague 8, Czech Republic; capekj@fzu.cz (J.Č.); Pincik789@gmail.com (J.P.)

**Keywords:** biomaterials, metallic composites, powder technology, zinc

## Abstract

Zinc-based alloys represent one of the most highly developed areas regarding biodegradable materials. Despite this, some general deficiencies such as cytotoxicity and poor mechanical properties (especially elongation), are not properly solved. In this work, a Zn-5Mg (5 wt.% Mg) composite material with tailored mechanical and superior corrosion properties is prepared by powder metallurgy techniques. Pure Zn and Mg are mixed and subsequently compacted by extrusion at 200 °C and an extrusion ratio of 10. The final product possesses appropriate mechanical properties (tensile yield strength = 148 MPa, ultimate tensile strength = 183 MPa, and elongation = 16%) and decreased by four times the release of Zn in the initial stage of degradation compared to pure Zn, which can highly decrease cytotoxicity effects and therefore positively affect the initial stage of the healing process.

## 1. Introduction

Binary zinc–magnesium alloys have been studied with regard to the improvement of corrosion protection of steel [1,2,3], and the development of arterial stents [4,5,6,7,8,9,10] and various biodegradable fixation devices [11,12]. As a relatively new biodegradable material, they can compete with magnesium products [7,8,9,10]. Although zinc (ρ = 7.14 g·cm^−3^) is much heavier than Mg (ρ = 1.74 g·cm^−3^), it is less susceptible to corrosion in the human environment. Various Zn-based materials with superior mechanical and corrosion properties have been developed and studied [9,10,13,14] but pure Zn (even in low doses) is relatively toxic to cells [15] compared to Mg. The daily zinc requirement for an adult is estimated to be 15 mg/day [7,16]. On the contrary, even up to 700 mg/day of Mg can be tolerated [7,16]. The degradation of Zn-based materials is connected to the production of Zn^2+^ ions, which are released to the surrounding extracellular space and spread into different parts of the organism. Zn^2+^ ions play an important role in various cellular processes (cell proliferation, differentiation, and signaling). Among other things, a high concentration of Zn^2+^ can induce apoptosis or necrosis or destroy ion-dependent intracellular signaling pathways [14]. On the contrary, zinc ions in adequate doses can enhance regulation of genes, cell survival/growth and differentiation, extracellular matrix (ECM) mineralization, and osteogenesis [17]. Magnesium is well tolerated by the organism in larger doses than zinc and affects activation of many enzymes, co-regulation of protein synthesis and muscle contraction, and stabilization of DNA and RNA [7]. Although the results of in vitro cytotoxicity tests are generally inconsistent [10,15,18] and long term in vivo tests do possess some good results [10], the lower initial release of Zn from the degradable implant is desirable to improve the osseointegration process during the initial stage of healing and suppress the possible formation of necrotic tissue. Unfortunately, alloying generally leads to a minor decrease in the corrosion rate of Zn-based alloys [10,14]. In this work, we selected a non-traditional way of producing a Zn-5Mg composite material by powder metallurgical methods. Although powder metallurgy is a powerful method for the production of materials with superior mechanical and corrosion properties, it has been very rarely used to prepare biodegradable Zn-based materials intended for medical devices [19,20]. However, our presented results demonstrate that this technique enables the preparation of materials with tailored mechanical and corrosion properties and improved biocompatibility.

## 2. Materials and Methods

### 2.1. Sample Preparation

Powders of pure Mg in the form of spherical particles 50–200 µm in diameter and pure Zn in the form of elongated irregular particles about 30–50 µm in thickness and 60–300 µm in length were mixed in Turbula^®^ T2F (WAB-GROUP, Muttenz, Switzerland) for 20 min in a ratio equal to the composition of Zn-5Mg (5 wt.% Mg). The mixture was subsequently compressed in a mold 20 mm in diameter by loading 80 kN for 2 min. Green compacts were extruded at 200 °C and an extrusion velocity of 5 mm/min. The final diameter of the extruded rod was 6.5 mm, which corresponds to an extrusion ratio equal to 10. All processing steps were performed under an air atmosphere. Pure Zn was prepared in the same way as the reference material.

### 2.2. Microstructure Characterization

Mechanical grinding and polishing were used for surface pre-treatment. Final polishing was performed using an Eposil F suspension (Metalco Testing s.r.o, Roztoky u Prahy, Czech Republic). The microstructure was characterized by optical microscopy and scanning electron microscopy (TescanVEGA3, TESCAN Brno, s.r.o, Brno, Czech Republic) equipped with Energy-Dispersive X-Ray Spectroscopy-EDS (AZtec, Oxford Instruments, Abingdon, United Kingdom).

### 2.3. Mechanical Properties

Vickers Hardness HV1 was measured on sample planes perpendicular to the extrusion direction. Compression tests were measured on cylindrical samples 5 mm in diameter and 7 mm in height. Classical dog bone specimens with gauge length equal to 10 mm and diameter equal to 4.5 mm were used for tensile tests. Shoulders on the side of specimens were 15 mm in length and 5.5 mm in diameter. The radius between the shoulders and loaded part was 3.5 mm. Both compressive and tensile tests were performed using a LabTest 5.250SP1-VM universal loading machine (LABORTECH s.r.o., Opava, Czech Republic) at a strain rate of 0.001 s^−1^. Loading was performed parallel to the extrusion direction. Tensile yield strength (TYS), ultimate tensile strength (UTS), and elongation to fracture were evaluated from engineering stress–strain curves.

### 2.4. Corrosion Properties

Samples 5 mm in diameter and 7 mm in height were immersed in simulated body fluid (SBF27) prepared according to Müller at al. [11] for 336 h. The ratio between sample surface and volume of solution was 100 mL·cm^−2^. After immersion testing corrosion products were removed from the samples by repeated immersion of the samples in a solution containing 200 g·L^−1^ CrO_3_. This process was performed until the change of the sample weight between steps was less than 0.001 g. Corrosion rates were calculated from the weight changes between the initial sample and dry sample with removed corrosion products and the concentration of released Zn ions into the corrosion media was determined by ICP-MS analyses (Elan DRC-e, Perkin-Elmer, Waltham, MA, USA). Cathodic curves with a general three electrode setup (sample = working electrode, glassy carbon = counter electrode, and Ag/AgCl containing saturated KCl = reference electrode) were measured using Parstat 3000-MC (AMETEK—Measurement, Communications & Testing, Berwyn, Pennsylvania, USA) in SBF. A sample with the same dimensions as for immersion tests mounted through the screw-thread to the Teflon holder was left in the corrosion media for 60 min to stabilize the open circuit potential (OCP); subsequently, polarization started from +0.02 versus OCP and continued to −0.6 versus OCP with a rate of 1 mV·s^−1^. Before all corrosion tests, samples were ground on SiC papers of up to P2000 and degreased in ethanol.

## 3. Results and Discussion

### 3.1. Microstructure Characterization

**Figure 1:**
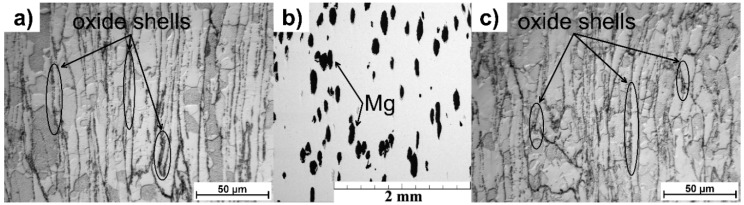
The microstructure of (**a**) Zn; (**b**) and (**c**) general view and detail of Zn-5Mg material prepared by powder metallurgy, respectively.

**Figure 2:**
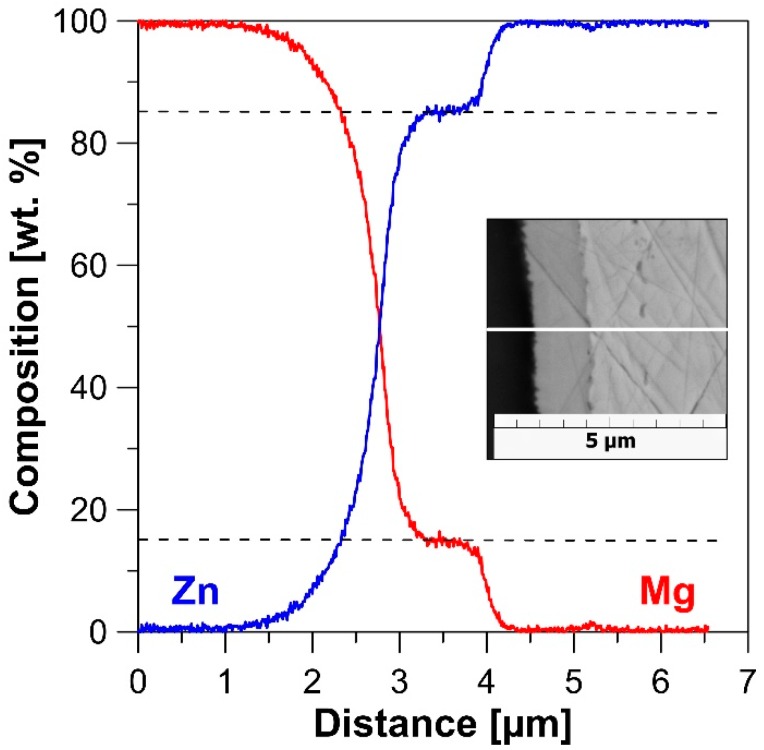
Line-scan of chemical composition at the interface of Zn and Mg.

The microstructure of the extruded materials is depicted in Figure 1. Both Zn (Figure 1a) and Zn-5Mg (Figure 1b,c) can be seen to be characterized by a partially recrystallized bimodal microstructure containing equiaxed grains with grain sizes in the range 10–30 µm and elongated grains 10 µm in thickness and 50–300 µm in length. Oxide shells, which come from the original powder surface, can be observed to surround the deformed particles. No systematic difference between the microstructure of the Zn matrix was observed for pure Zn and Zn-5Mg extruded materials. Magnesium particles (black areas in Figure 1) can be seen to be homogeneously distributed in the Zn matrix. The interface between Zn and magnesium is occupied by an Mg_2_Zn_11_ intermetallic phase [21] (Figure 2) which belongs to the gamma brasses type of phase and contains 15.5 wt. % of Mg. This thermodynamically stable intermetallic phase [22] is generally formed during solidification of Zn–Mg alloys as part of the eutectic reaction. The average thickness of the presented phase was 1.2 ± 0.2 µm. Yang et. al. [23] have also studied Zn–Mg composite materials prepared from pure powders by spark plasma sintering (SPS). Final composites were composed of a mixture of stable Mg_2_Zn_11_ and metastable Mg_2_Zn. In addition, no pure magnesium remained in the microstructure [23].

### 3.2. Mechanical Properties

**Figure 3:**
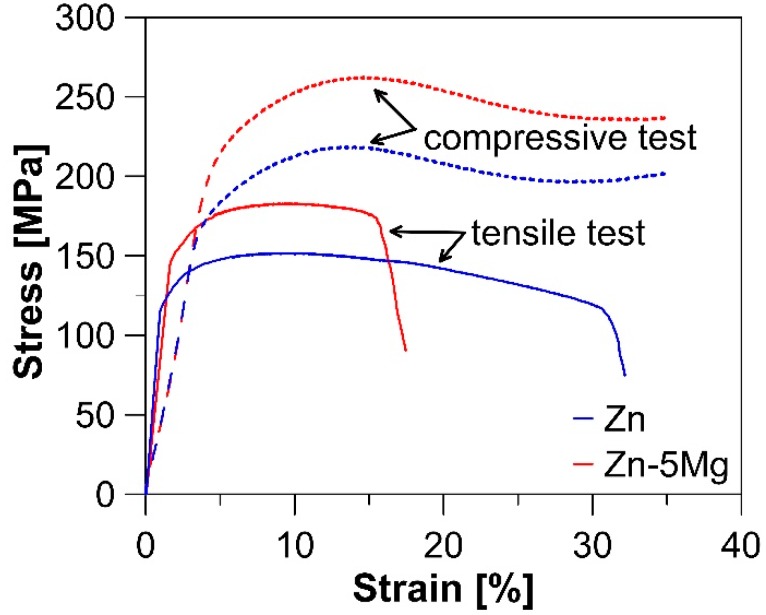
Compressive and tensile stress–strain curves for extruded Zn and Zn-5Mg.

Tensile and compressive curves of Zn and Zn-5Mg and evaluated values of mechanical properties are shown in Figure 3 and Table 1, respectively. Due to similarities in the microstructure of the Zn matrix, the difference in mechanical behavior can be attributed to the addition of Mg particles. This improves the yield stress (TYS—tensile yield strength, CYS—compressive yield strength) and ultimate stress (UTS—ultimate tensile strength, UCS—ultimate compressive strength) values under both tension and compression loading. The general increase in presented values is about 40 MPa. Such an improvement is related to the presence of an Mg_2_Zn_11_ intermetallic phase formed on the interface between the Mg and Zn particles (Figure 2). On the contrary, the specified phase is brittle and causes a partial decrease in the ductility. Nevertheless, both Zn and Zn-5Mg are still characterized by excellent elongation (35 ± 4% and 16 ± 2%, respectively) compared to various Zn-based alloys [10]. Yang et al. [23] measured CYS for Zn-5Mg (186.63 ± 26.54 MPa) prepared by SPS and indicate the possible use of these materials for some applications without load-bearing requirements. However, the values of 148 ± 6 MPa for TYS and 183 ± 4 MPa for UTS measured for extruded Zn-5Mg are not far from generally required values for fixation medical devices (TYS > 200 MPa, UTS > 270 MPa), and suitable processing of these materials could lead to the desired values. Additionally, the value of modulus of elasticity (E) is slightly lower for Zn-5Mg (81 GPa) as a consequence of the lower value of E for pure Mg (45 GPa) compared to pure Zn (108 GPa). Such behavior is also desirable to prevent the stress shielding effect.

### 3.3. Corrosion Properties

**Figure 4:**
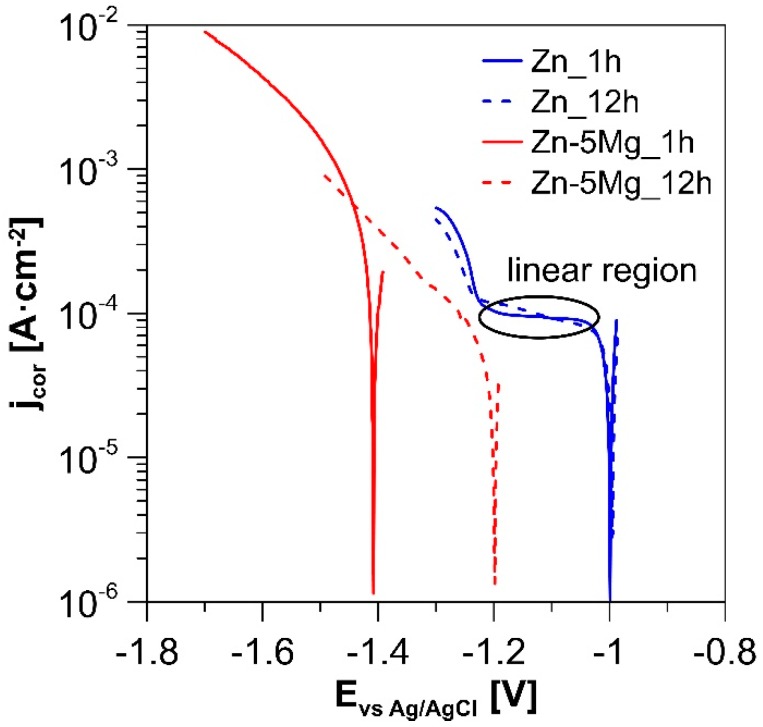
Polarization curves for extruded Zn and Zn-5Mg.

Corrosion rates evaluated from weight changes were 0.373 ± 0.004 and 0.043 ± 0.020 mg·cm^−2^·day^−1^ for Zn and Zn-5Mg respectively. Evaluation from Zn^2+^ concentration led to lower values, namely to 0.082 ± 0.016 and 0.022 ± 0.010 mg·cm^−2^·day^−1^, due to the presence of Zn in the solid state of corrosion products. This result reveals a surprising four-time decrease in released Zn^2+^ ions for Zn-5Mg. In fact, during in vitro cytotoxicity testing, even 50% dilution generally highly improves cell viability [15]. Regarding the alloying of Zn-based biodegradable alloys, such a huge change in corrosion rate has never been observed, to the best of our knowledge. To explain this behavior, potentiodynamic curves were measured after 1 and 12 h of immersion in SBF. The curves representing the polarization of pure Zn (blue lines in Figure 4) can be seen to be similar after 1 and 12 h in SBF. Both curves contain a specific linear region which indicates a cathodic reaction controlled by the diffusion of oxygen. The Ecor value of Zn-5Mg (−1.42 V) is due to the dissolution of less noble Mg, producing a more negative value compared to the E_cor_ of pure Zn (−0.98 V). At this potential, the reduction of water (Equation (1)) accompanied by hydrogen release is a dominant cathodic reaction.

2H_2_O + 2e^−^ → H_2_ + 2OH^−^ (1)

Magnesium works as a sacrificial anode and helps to protect the zinc matrix. Consequently, the decrease in current densities on the measured cathodic curve of sample Zn-5Mg immersed for 12 h was observed. The observed behavior caused the decrease in corrosion rate of the alloy during longer immersion tests and also lowered the amount of released Zn^2+^ ions during the initial stage of the corrosion process, which can particularly suppress the cytotoxic effect and improve the material biocompatibility. In contrary to the present study, Yang et al. [23] observed an increased corrosion rate for Zn-5Mg compared to pure Zn. They claimed that the corrosion rate of Zn-5Mg was increased as a consequence of micro-galvanic cells among MgZn_2_, Mg_2_Zn_11_, and pure Zn. In this study, the intermetallic phase occupied the only low area at the interface of pure Zn and Mg. Pure magnesium is even less noble than the presented phases, works as strong sacrificial anode, and enables the passivation of Zn. This leads in particular to the lower corrosion rates of the Zn-5Mg composite in this specific case and supports the idea that variation in phase composition of these types of materials can highly affect corrosion behavior.

## 4. Conclusions

This paper introduces a novel approach for the preparation of Zn-based biodegradable materials using the powder metallurgy technique. In this work, a Zn-5Mg alloy was successfully prepared by extrusion of a green compact containing a mixture of pure Zn and Mg powders. Compared to the product made using Zn powder, the addition of Mg was observed to positively affect TYS and UTS values while maintaining excellent elongation. Additionally, Zn-5Mg was characterized by a decreased value of modulus of elasticity, which is highly desirable for fixation devices intended for traumatology applications, and a lower corrosion rate compared to pure Zn and Zn-based alloys. A lower amount of Zn ions released by the corrosion process can suppress cytotoxicity effects. Generally, the observed behaviour will lead to a significant improvement in the biocompatibility of the material.

## Figures and Tables

**Table 1:** Tensile and compressive mechanical properties of Zn and Zn-5Mg materials after compaction by extrusion at 200 °C. Compressive tests were finished at 35% of relative deformation due to the absence of fracture.

	HV1	Tensile Test			Compression Test	
		TYS (0.2 Proof Stress) (MPa)	UTS (MPa)	Elongation (%)	CYS (0.2 Proof Stress) (MPa)	UCS (MPa)
**Zn**	35	114 ± 5	156 ± 5	35 ± 4	170 ± 6	215 ± 4
**Zn-5Mg**	36 (Zn) 51 (Mg)	148 ± 6	183 ± 4	16 ± 2	209 ± 6	256 ± 6
